# Supplementary material for: Adaptation of hepatitis C virus to interferon lambda polymorphism across multiple viral genotypes
Source: eLife. 2019 Sep 3;8:e42542. doi: 10.7554/eLife.42542 (PMC6721370; doi:10.7554/eLife.42542)
Supplement: Supplementary file 1. — HCV genes and positions on the HCV proteome are given in the first and the second column of the table. Amino acid residuals on the associated positions are given in the second column. [file elife-42542-supp1.docx]

**HCV amino acid positions with significant association p-values from genome-to-genome analysis (column 3), viral load GWAS analysis (column 4) and viral load residual GWAS analysis (column 5), for viral genotype 1a. HCV genes and positions on the HCV proteome are given in the first and the second column of the table. Amino acid residuals on the associated positions are given in the second column.**

| **HCV genes** | **Position  (amino acids)** | **G2G p-values** | **Viral load  GWAS p-values** | **Viral load residual GWAS p-values** |
| --- | --- | --- | --- | --- |
| NS3 | 1612(T) | 1.54e-08 (OR 1.11; beta 0.11; 97%CI 1.07-1.15) | 1.42e-10 (r2 0.127) | 1.35e-08 (r2 0.0477) |
| NS5A | 2024(V) | 5.81e-09 (OR 1.04; beta 0.038; 97%CI 1.03-1.05) | 5.96e-14 (r2 0.117) | 2.37e-12 (r2 0.0158) |
| NS5A | 2252(I) | 1.29e-25 (OR 1.12; beta 0.11; 97%CI 1.1-1.15) | 1.18e-21 (r2 0.129) | 3.15e-18 (r2 0.0252) |
| NS5A | 2252(V) | 1.72e-22 (OR 0.889; beta -0.12; 97%CI 0.868-0.91) | 2.83e-16 (r2 0.123) | 1.67e-13 (r2 0.0191) |
| NS5A | 2287(I) | 1.54e-14 (OR 1.09; beta 0.088; 97%CI 1.07-1.12) | 4.38e-09 (r2 0.114) | 1.76e-07 (r2 0.0114) |
| NS5A | 2298(V) | 1.66e-14 (OR 0.917; beta -0.086; 97%CI 0.897-0.938) | 4.97e-08 (r2 0.113) | 1.52e-06 (r2 0.0102) |
| NS5A | 2372(S) | 1.63e-14 (OR 1.06; beta 0.054; 97%CI 1.04-1.07) | 2.54e-08 (r2 0.113) | 8.48e-07 (r2 0.0105) |

*Number of samples: 3548. G2G (Genome to genome) analysis (column 3) was performed using a logistic regression between binary viral amino acid variables as train of interest, depicting the presence or absence of an amino acid, and host SNP. Viral load GWAS analysis (column 4) was performed using linear regression, between transformed viral load as trait of interest and viral amino acid variations. Viral load residual analysis (column 5) was performed using linear regression, between viral load residuals, obtained after regressing transformed viral load over the host SNP, as trait of interest and viral amino acid variations. All three analyses were corrected for host and viral stratification by adding sex, country of origin, self-reported ethnicity, cirrhosis status, prior treatment experience and first 5 viral phylogenetic principal components as covariates.
