# Supplementary material for: Adaptation of hepatitis C virus to interferon lambda polymorphism across multiple viral genotypes
Source: eLife. 2019 Sep 3;8:e42542. doi: 10.7554/eLife.42542 (PMC6721370; doi:10.7554/eLife.42542)
Supplement: Supplementary file 3. — The table consists of significant p-values and NA represents non-significant p-values. [file elife-42542-supp3.docx]

**Genome to genome analysis results for Asians and Europeans. The table consists of significant p-values and NA represents non-significant p-values.**

| **HCV genes** | **Position**  **(amino acid)** | **Asians** | **Europeans** |
| --- | --- | --- | --- |
| NS3 | 1332(A) | NA | 6.37e-12 (OR 1.04; beta 0.04; 97%CI 1.03-1.05) |
| NS3 | 1355(I) | NA | 3.54e-06 (OR 1.06; beta 0.054; 97%CI 1.03-1.08) |
| NS3 | 1370(I) | NA | 1.86e-07 (OR 0.963; beta -0.037; 97%CI 0.95-0.977) |
| NS3 | 1473(D) | NA | 2.41e-08 (OR 1.03; beta 0.026; 97%CI 1.02-1.04) |
| NS3 | 1612(I) | NA | 1.51e-09 (OR 0.925; beta -0.078; 97%CI 0.901-0.948) |
| NS3 | 1612(N) | NA | 5.53e-10 (OR 1.05; beta 0.045; 97%CI 1.03-1.06) |
| NS3 | 1612(T) | NA | 4.8e-10 (OR 1.08; beta 0.079; 97%CI 1.06-1.11) |
| NS4A | 1671(T) | NA | 7e-10 (OR 1.03; beta 0.026; 97%CI 1.02-1.03) |
| NS4A | 1703(R) | NA | 2.5e-06 (OR 1.06; beta 0.059; 97%CI 1.04-1.09) |
| NS5A | 1996(R) | NA | 6.36e-07 (OR 1.01; beta 0.009; 97%CI 1.01-1.01) |
| NS5A | 2034(D) | NA | 1.26e-09 (OR 1.02; beta 0.018; 97%CI 1.01-1.02) |
| NS5A | 2040(K) | NA | 2.14e-06 (OR 0.987; beta -0.014; 97%CI 0.981-0.992) |
| NS5A | 2040(R) | NA | 5.49e-07 (OR 1.02; beta 0.016; 97%CI 1.01-1.02) |
| NS5A | 2047(A) | NA | 5.42e-13 (OR 1.04; beta 0.037; 97%CI 1.03-1.05) |
| NS5A | 2065(H) | NA | 7.32e-13 (OR 1.03; beta 0.027; 97%CI 1.02-1.04) |
| NS5A | 2080(K) | 1.92e-10 (OR 1.15; beta 0.14; 97%CI 1.1-1.2) | 1.94e-09 (OR 1.01; beta 0.015; 97%CI 1.01-1.02) |
| NS5A | 2209(L) | NA | 2.77e-11 (OR 1.02; beta 0.017; 97%CI 1.01-1.02) |
| NS5A | 2209(P) | NA | 4.08e-10 (OR 0.98; beta -0.02; 97%CI 0.974-0.986) |
| NS5A | 2211(L) | NA | 1.84e-09 (OR 0.988; beta -0.012; 97%CI 0.985-0.992) |
| NS5A | 2214(A) | NA | 4.16e-07 (OR 1.02; beta 0.016; 97%CI 1.01-1.02) |
| NS5A | 2214(T) | NA | 8.98e-08 (OR 0.984; beta -0.016; 97%CI 0.978-0.99) |
| NS5A | 2224(L) | 8.16e-07 (OR 1.07; beta 0.069; 97%CI 1.04-1.1) | 1.07e-06 (OR 1.01; beta 0.0067; 97%CI 1-1.01) |
| NS5A | 2234(W) | 4.06e-06 (OR 1.09; beta 0.082; 97%CI 1.05-1.12) | NA |
| NS5A | 2237(K) | 1.61e-06 (OR 1.08; beta 0.077; 97%CI 1.05-1.11) | NA |
| NS5A | 2251(I) | 1.79e-07 (OR 1.11; beta 0.1; 97%CI 1.07-1.15) | NA |
| NS5A | 2252(I) | NA | 1.22e-22 (OR 1.07; beta 0.067; 97%CI 1.05-1.08) |
| NS5A | 2252(V) | NA | 2.38e-21 (OR 0.931; beta -0.071; 97%CI 0.918-0.945) |
| NS5A | 2287(I) | NA | 2.3e-13 (OR 1.05; beta 0.051; 97%CI 1.04-1.07) |
| NS5A | 2287(V) | 4.71e-07 (OR 0.883; beta -0.12; 97%CI 0.842-0.927) | 3.26e-10 (OR 0.951; beta -0.05; 97%CI 0.936-0.966) |
| NS5A | 2298(L) | NA | 4.6e-06 (OR 1.04; beta 0.036; 97%CI 1.02-1.05) |
| NS5A | 2298(V) | NA | 2.04e-12 (OR 0.957; beta -0.044; 97%CI 0.946-0.969) |
| NS5A | 2300(P) | 2.76e-07 (OR 1.14; beta 0.13; 97%CI 1.08-1.19) | 3.63e-06 (OR 1.02; beta 0.019; 97%CI 1.01-1.03) |
| NS5A | 2300(S) | NA | 8.57e-07 (OR 0.983; beta -0.017; 97%CI 0.977-0.99) |
| NS5A | 2318(S) | NA | 1.03e-06 (OR 1.01; beta 0.013; 97%CI 1.01-1.02) |
| NS5A | 2320(K) | NA | 7.82e-07 (OR 0.974; beta -0.026; 97%CI 0.964-0.984) |
| NS5A | 2320(Q) | NA | 6.9e-07 (OR 1.03; beta 0.034; 97%CI 1.02-1.05) |
| NS5A | 2358(P) | NA | 1.74e-08 (OR 1.02; beta 0.016; 97%CI 1.01-1.02) |
| NS5A | 2360(A) | NA | 7.15e-12 (OR 1.03; beta 0.027; 97%CI 1.02-1.04) |
| NS5A | 2372(S) | NA | 5.75e-07 (OR 1.03; beta 0.027; 97%CI 1.02-1.04) |
| NS5A | 2385(C) | NA | 4.67e-24 (OR 1.07; beta 0.069; 97%CI 1.06-1.09) |
| NS5A | 2385(H) | NA | 9.8e-07 (OR 1.01; beta 0.012; 97%CI 1.01-1.02) |
| NS5A | 2385(Y) | NA | 1.29e-22 (OR 0.938; beta -0.064; 97%CI 0.926-0.95) |
| NS5A | 2413(S) | 1.97e-06 (OR 0.889; beta -0.12; 97%CI 0.847-0.933) | NA |
| NS5A | 2414(K) | NA | 1.85e-07 (OR 1.02; beta 0.016; 97%CI 1.01-1.02) |
| NS5A | 2414(T) | NA | 2.96e-09 (OR 0.964; beta -0.037; 97%CI 0.952-0.976) |
| NS5A | 2416(D) | NA | 1.33e-06 (OR 0.984; beta -0.016; 97%CI 0.978-0.991) |
| NS5A | 2416(G) | NA | 1.81e-12 (OR 1.03; beta 0.028; 97%CI 1.02-1.04) |
| NS5A | 2416(N) | NA | 1.62e-10 (OR 1.02; beta 0.024; 97%CI 1.02-1.03) |
| NS5A | 2416(S) | NA | 4.03e-14 (OR 0.973; beta -0.027; 97%CI 0.966-0.98) |
| NS5A | 2420(N) | NA | 2.77e-08 (OR 1.02; beta 0.019; 97%CI 1.01-1.03) |
| NS5B | 2510(N) | NA | 5.76e-07 (OR 1.01; beta 0.011; 97%CI 1.01-1.02) |
| NS5B | 2567(I) | NA | 1.87e-13 (OR 1.03; beta 0.031; 97%CI 1.02-1.04) |
| NS5B | 2567(M) | NA | 5.26e-07 (OR 1.01; beta 0.014; 97%CI 1.01-1.02) |
| NS5B | 2567(V) | NA | 1.02e-08 (OR 0.975; beta -0.025; 97%CI 0.967-0.984) |
| NS5B | 2570(T) | NA | 4.16e-09 (OR 1.02; beta 0.024; 97%CI 1.02-1.03) |
| NS5B | 2570(V) | NA | 7.05e-19 (OR 0.958; beta -0.043; 97%CI 0.948-0.967) |
| NS5B | 2576(A) | NA | 3.43e-39 (OR 1.05; beta 0.052; 97%CI 1.05-1.06) |
| NS5B | 2576(P) | NA | 1.08e-46 (OR 0.944; beta -0.058; 97%CI 0.936-0.951) |
| NS5B | 2576(S) | NA | 7.54e-13 (OR 1.02; beta 0.021; 97%CI 1.02-1.03) |
| NS5B | 2632(K) | NA | 1.8e-06 (OR 0.977; beta -0.023; 97%CI 0.968-0.986) |
| NS5B | 2729(Q) | NA | 2.44e-18 (OR 0.941; beta -0.061; 97%CI 0.928-0.954) |
| NS5B | 2729(R) | NA | 1.82e-22 (OR 1.08; beta 0.072; 97%CI 1.06-1.09) |
| NS5B | 2773(A) | NA | 4.13e-08 (OR 0.989; beta -0.011; 97%CI 0.985-0.993) |
| NS5B | 2794(Q) | NA | 3.65e-09 (OR 1.02; beta 0.017; 97%CI 1.01-1.02) |
| NS5B | 2834(Q) | NA | 1.94e-06 (OR 0.996; beta -0.0042; 97%CI 0.994-0.998) |
| NS5B | 2839(L) | NA | 8.01e-07 (OR 1.01; beta 0.011; 97%CI 1.01-1.02) |
| NS5B | 2858(Q) | NA | 2.45e-07 (OR 1.01; beta 0.0087; 97%CI 1.01-1.01) |
| NS5B | 2860(G) | 6.76e-07 (OR 1.06; beta 0.059; 97%CI 1.04-1.09) | 2.44e-07 (OR 1.01; beta 0.011; 97%CI 1.01-1.02) |
| NS5B | 2893(S) | NA | 2.18e-06 (OR 1.01; beta 0.01; 97%CI 1.01-1.01) |
| NS5B | 2893(T) | NA | 1.6e-08 (OR 0.986; beta -0.014; 97%CI 0.981-0.991) |
| NS5B | 2937(R) | NA | 1.22e-06 (OR 1.03; beta 0.032; 97%CI 1.02-1.05) |
| NS5B | 2986(H) | NA | 2.26e-07 (OR 0.958; beta -0.043; 97%CI 0.943-0.974) |
| NS5B | 2986(R) | NA | 3.45e-06 (OR 1.04; beta 0.038; 97%CI 1.02-1.06) |
| NS5B | 2991(H) | NA | 5.41e-10 (OR 0.972; beta -0.028; 97%CI 0.963-0.981) |
| NS5B | 2991(Y) | NA | 7.39e-15 (OR 1.04; beta 0.038; 97%CI 1.03-1.05) |
| NS5B | 3008(F) | NA | 4.88e-07 (OR 1.01; beta 0.0067; 97%CI 1-1.01) |

*Number of Asian samples: 1103. Number of European samples: 6704. G2G (Genome to genome) analysis for Europeans as well as Asians was performed using a logistic regression between binary viral amino acid variables as train of interest, depicting the presence or absence of an amino acid, and host SNP. All analyses were corrected for host and viral stratification by adding sex, country of origin, self-reported ethnicity, cirrhosis status, prior treatment experience and first 5 viral phylogenetic principal components as covariates.
