# Supplementary material for: Adaptation of hepatitis C virus to interferon lambda polymorphism across multiple viral genotypes
Source: eLife. 2019 Sep 3;8:e42542. doi: 10.7554/eLife.42542 (PMC6721370; doi:10.7554/eLife.42542)
Supplement: Supplementary file 4. — The table consists of significant p-values and NA represents non-significant p-values. [file elife-42542-supp4.docx]

**Genome to genome analysis results per genotype in European samples. The table consists on significant p-values and NA represents non-significant p-values.**

| **HCV genes** | **Position (amino acids)** | **genotype 1a**  **(N = 2987)** | **genotype 1b**  **(N = 1133)** | **genotype 2a**  **(N = 100)** | **genotype 2b**  **(N = 421)** | **genotype 3a**  **(N = 1635)** | **genotype 4a**  **(N = 178)** |
| --- | --- | --- | --- | --- | --- | --- | --- |
| NS3 | 1332(A) | 6.73e-11 (OR 1.06; beta 0.063; 97%CI 1.04-1.08) | NA | NA | NA | NA | NA |
| NS3 | 1355(I) | 9.32e-07 (OR 1.1; beta 0.098; 97%CI 1.06-1.15) | NA | NA | NA | NA | NA |
| NS3 | 1370(I) | NA | 3.99e-07 (OR 0.838; beta -0.18; 97%CI 0.784-0.897) | NA | NA | NA | NA |
| NS3 | 1473(D) | 5.88e-07 (OR 1.03; beta 0.033; 97%CI 1.02-1.05) | NA | NA | NA | NA | NA |
| NS3 | 1612(I) | 4.89e-14 (OR 0.862; beta -0.15; 97%CI 0.83-0.896) | NA | NA | NA | NA | NA |
| NS3 | 1612(N) | 2.69e-10 (OR 1.08; beta 0.081; 97%CI 1.06-1.11) | NA | NA | NA | NA | NA |
| NS3 | 1612(T) | 5.44e-08 (OR 1.11; beta 0.11; 97%CI 1.07-1.16) | NA | NA | NA | NA | NA |
| NS4A | 1671(T) | 1.15e-07 (OR 1.04; beta 0.035; 97%CI 1.02-1.05) | NA | NA | NA | NA | NA |
| NS4A | 1683(I) | 1.95e-06 (OR 1.03; beta 0.027; 97%CI 1.02-1.04) | NA | NA | NA | NA | NA |
| NS4A | 1703(R) | NA | 1.67e-06 (OR 1.2; beta 0.18; 97%CI 1.12-1.29) | NA | NA | NA | NA |
| NS5A | 1996(R) | 2.6e-07 (OR 1.02; beta 0.016; 97%CI 1.01-1.02) | NA | NA | NA | NA | NA |
| NS5A | 2009(I) | 3.31e-06 (OR 1.02; beta 0.018; 97%CI 1.01-1.03) | NA | NA | NA | NA | NA |
| NS5A | 2024(V) | 3.39e-07 (OR 1.04; beta 0.036; 97%CI 1.02-1.05) | NA | NA | NA | NA | NA |
| NS5A | 2034(T) | NA | NA | NA | NA | 3.48e-07 (OR 0.903; beta -0.1; 97%CI 0.868-0.939) | NA |
| NS5A | 2047(A) | 3.22e-17 (OR 1.08; beta 0.074; 97%CI 1.06-1.1) | NA | NA | NA | NA | NA |
| NS5A | 2065(H) | 1.3e-06 (OR 1.02; beta 0.015; 97%CI 1.01-1.02) | NA | NA | NA | NA | NA |
| NS5A | 2080(K) | NA | 1.07e-09 (OR 1.08; beta 0.081; 97%CI 1.06-1.11) | NA | NA | NA | NA |
| NS5A | 2211(L) | 4.43e-06 (OR 0.986; beta -0.014; 97%CI 0.98-0.992) | NA | NA | NA | NA | NA |
| NS5A | 2224(L) | NA | 1.03e-06 (OR 1.04; beta 0.035; 97%CI 1.02-1.05) | NA | NA | NA | NA |
| NS5A | 2252(I) | 1.29e-24 (OR 1.13; beta 0.12; 97%CI 1.1-1.16) | NA | NA | NA | NA | NA |
| NS5A | 2252(V) | 5.67e-22 (OR 0.881; beta -0.13; 97%CI 0.859-0.904) | NA | NA | NA | 2.28e-06 (OR 0.946; beta -0.055; 97%CI 0.925-0.968) | NA |
| NS5A | 2266(V) | NA | NA | NA | NA | 1.11e-06 (OR 0.921; beta -0.082; 97%CI 0.891-0.952) | NA |
| NS5A | 2287(I) | 1.23e-10 (OR 1.08; beta 0.079; 97%CI 1.06-1.11) | NA | NA | NA | NA | NA |
| NS5A | 2287(V) | 1.19e-08 (OR 0.924; beta -0.079; 97%CI 0.9-0.95) | NA | NA | NA | NA | NA |
| NS5A | 2298(V) | 1.53e-13 (OR 0.914; beta -0.089; 97%CI 0.893-0.936) | NA | NA | NA | NA | NA |
| NS5A | 2300(P) | NA | 1.11e-09 (OR 1.1; beta 0.098; 97%CI 1.07-1.14) | NA | NA | NA | NA |
| NS5A | 2300(S) | NA | 9.84e-07 (OR 0.934; beta -0.069; 97%CI 0.908-0.959) | NA | NA | NA | NA |
| NS5A | 2320(Q) | 2.99e-09 (OR 1.09; beta 0.084; 97%CI 1.06-1.12) | NA | NA | NA | NA | NA |
| NS5A | 2360(A) | NA | 2.2e-09 (OR 1.13; beta 0.12; 97%CI 1.08-1.17) | NA | NA | NA | NA |
| NS5A | 2371(S) | 3.06e-06 (OR 1.03; beta 0.028; 97%CI 1.02-1.04) | NA | NA | NA | NA | NA |
| NS5A | 2372(S) | 6.8e-12 (OR 1.05; beta 0.053; 97%CI 1.04-1.07) | NA | NA | NA | NA | NA |
| NS5A | 2385(C) | 1.59e-15 (OR 1.11; beta 0.1; 97%CI 1.08-1.13) | 3.4e-06 (OR 1.05; beta 0.048; 97%CI 1.03-1.07) | NA | NA | NA | NA |
| NS5A | 2385(Y) | 1.29e-14 (OR 0.913; beta -0.091; 97%CI 0.892-0.934) | NA | NA | NA | NA | NA |
| NS5A | 2414(K) | 1.62e-07 (OR 1.03; beta 0.033; 97%CI 1.02-1.05) | NA | NA | NA | NA | NA |
| NS5A | 2414(T) | 6.62e-09 (OR 0.929; beta -0.074; 97%CI 0.906-0.952) | NA | NA | NA | NA | NA |
| NS5A | 2416(G) | NA | NA | NA | NA | 6.4e-08 (OR 1.08; beta 0.072; 97%CI 1.05-1.1) | NA |
| NS5A | 2416(N) | NA | NA | NA | NA | 1.47e-06 (OR 1.08; beta 0.081; 97%CI 1.05-1.12) | NA |
| NS5A | 2416(S) | NA | NA | NA | NA | 9.39e-12 (OR 0.882; beta -0.12; 97%CI 0.852-0.914) | NA |
| NS5A | 2420(N) | NA | NA | NA | NA | 3.24e-08 (OR 1.08; beta 0.077; 97%CI 1.05-1.11) | NA |
| NS5B | 2510(N) | 7.46e-07 (OR 1.02; beta 0.024; 97%CI 1.01-1.03) | NA | NA | NA | NA | NA |
| NS5B | 2567(I) | 3.86e-14 (OR 1.03; beta 0.03; 97%CI 1.02-1.04) | NA | NA | NA | NA | NA |
| NS5B | 2570(T) | NA | NA | NA | NA | 1.25e-12 (OR 1.1; beta 0.098; 97%CI 1.07-1.13) | NA |
| NS5B | 2570(V) | NA | NA | NA | NA | 3.06e-17 (OR 0.844; beta -0.17; 97%CI 0.812-0.878) | NA |
| NS5B | 2576(A) | 1.77e-09 (OR 1.02; beta 0.017; 97%CI 1.01-1.02) | NA | NA | 2.31e-07 (OR 1.2; beta 0.18; 97%CI 1.12-1.28) | 8.23e-15 (OR 1.07; beta 0.068; 97%CI 1.05-1.09) | NA |
| NS5B | 2576(P) | 4.27e-08 (OR 0.983; beta -0.017; 97%CI 0.978-0.989) | NA | NA | 9.9e-14 (OR 0.767; beta -0.27; 97%CI 0.716-0.82) | 5.46e-12 (OR 0.946; beta -0.055; 97%CI 0.931-0.961) | 5.08e-07 (OR 0.826; beta -0.19; 97%CI 0.769-0.887) |
| NS5B | 2729(Q) | 6.07e-14 (OR 0.902; beta -0.1; 97%CI 0.878-0.927) | NA | NA | NA | NA | NA |
| NS5B | 2729(R) | 1.55e-13 (OR 1.11; beta 0.1; 97%CI 1.08-1.14) | NA | NA | NA | NA | NA |
| NS5B | 2794(Q) | NA | NA | NA | NA | 1.14e-09 (OR 1.08; beta 0.077; 97%CI 1.05-1.11) | NA |
| NS5B | 2844(I) | 9.14e-07 (OR 1.03; beta 0.033; 97%CI 1.02-1.05) | NA | NA | NA | NA | NA |
| NS5B | 2937(K) | 7.69e-07 (OR 0.945; beta -0.056; 97%CI 0.925-0.967) | NA | NA | NA | NA | NA |
| NS5B | 2937(R) | NA | NA | NA | NA | 2.68e-06 (OR 1.07; beta 0.069; 97%CI 1.04-1.1) | NA |
| NS5B | 2991(H) | NA | NA | NA | NA | 3.22e-10 (OR 0.883; beta -0.12; 97%CI 0.85-0.918) | NA |
| NS5B | 2991(Y) | NA | NA | NA | NA | 2.19e-15 (OR 1.17; beta 0.16; 97%CI 1.13-1.22) | NA |
| NS5B | 3008(F) | 1.02e-07 (OR 1.02; beta 0.016; 97%CI 1.01-1.02) | NA | NA | NA | NA | NA |

*Genome to genome analysis for all viral genotypes was performed using a logistic regression between binary viral amino acid variables as train of interest, depicting the presence or absence of an amino acid, and host SNP. All analyses were corrected for host and viral stratification by adding sex, country of origin, self-reported ethnicity, cirrhosis status, prior treatment experience and first 5 viral phylogenetic principal components as covariates.
